# An analytical solution for two-dimensional vacuum preloading combined with electro-osmosis consolidation using *EKG* electrodes

**DOI:** 10.1371/journal.pone.0180974

**Published:** 2017-08-03

**Authors:** Yang Shen, Chenchen Qiu, Yande Li, Wen Shi, Xiaoxi Rui

**Affiliations:** 1 Key Laboratory of Ministry of Education for Geo-mechanics and Embankment Engineering, Hohai University, Nanjing, China; 2 China Communications Construction Company Limited Tianjin Port & Waterway Prospection & Design Research institute Co., Lto. Tianjin, China; East China Normal University, CHINA

## Abstract

China is a country with vast territory, but economic development and population growth have reduced the usable land resources in recent years. Therefore, reclamation by pumping and filling is carried out in eastern coastal regions of China in order to meet the needs of urbanization. However, large areas of reclaimed land need rapid drainage consolidation treatment. Based on past researches on how to improve the treatment efficiency of soft clay using vacuum preloading combined with electro-osmosis, a two-dimensional drainage plane model was proposed according to the Terzaghi and Esrig consolidation theory. However, the analytical solution using two-dimensional plane model was never involved. Current analytical solutions can’t have a thorough theoretical analysis of practical engineering and give relevant guidance. Considering the smearing effect and the rectangle arrangement pattern, an analytical solution is derived to describe the behavior of pore-water and the consolidation process by using EKG (electro-kinetic geo synthetics) materials. The functions of EKG materials include drainage, electric conduction and corrosion resistance. Comparison with test results is carried out to verify the analytical solution. It is found that the measured value is larger than the applied vacuum degree because of the stacking effect of the vacuum preloading and electro-osmosis. The trends of the mean measured value and the mean analytical value processes are comparable. Therefore, the consolidation model can accurately assess the change in pore-water pressure and the consolidation process during vacuum preloading combined with electro-osmosis.

## Introduction

China has a vast territory, but the distribution of land is uneven. A large amount of population has gathered in eastern coastal regions, which significantly reduces the available land for economic development. Reclaiming land along the sea and beach is usually carried out in this area to solve this problem. The soft clay and dredged silt after reclamation are characterized by high water content and high compressibility but low shear strength. There are several ways for land reclamation, for example, the electro-osmotic dewatering method could drain water out of the low permeable soft clay quickly. However, due to the electro-chemical reaction, metal electrode would be corroded and of which has restricted its application [1]. The vacuum preloading could be used for foundation treatment with a large area of soft clay; a long period of construction was usually required. Hence, a second treatment needs to be carried out [2]. Thus, conventional preloading, vacuum preloading and electro-osmosis require a long period of construction and have low efficiency.

Due to the positive interaction between vacuum preloading and electro-osmosis in the cathode, the efficiency of foundation treatment can be remarkably improved. Current research on conventional vacuum preloading electro-osmosis mainly focuses on varying the applied potential, vacuum degree and intermittent electricity. With extensive research on this mechanism and some new electrode materials [3], emphasis has gradually shifted to the derivation of a consolidated analytical solution of vacuum preloading combined with electro-osmosis.

Considering the drainage in the cathode and non-drainage in the anode, the two-dimensional consolidation equation and analytical solution has been studied [4] according to the theory by Esrig [5] and Terzaghi. The one-dimensional analytical solution was derived using an axisymmetric model [6]. Considering the smearing effect, Li [7] derived an analytical solution for vacuum preloading combined with electro-osmosis for axisymmetric model.

Considering the effect of vacuum preloading in the boundary conditions [8–10], the consolidation theory of vacuum preloading combined with the electro-osmosis method was proposed according to the electro-osmotic consolidation theory [11–12]. During the test, the whole apparatus, including the electrodes, soil and power supply, was regarded as an electrolytic tank. The redox reaction would occur on the interface of the electrode and oil. So, the electrode corrosion cannot be avoided in the conventional electro-osmosis method, especially in the interface of the metal electrodes and the soil. As a result, there is always a wide gap between the analytical solution and measured values. Additionally, the drainage material was only set in the cathode, which is inconsistent with engineering practice.

The anode and cathode can be used as the transmission channel for vacuum transferring and drainage when EKG is applied [13–14]. Therefore, inhibition in the anode and promotion in the cathode can be produced as a result of the simultaneous treatments of vacuum preloading and electro-osmosis [15–18]. Moreover, the analytical solution using a two-dimensional plane model was never resolved. Current analytical solutions are not based on a thorough theoretical analysis of practical engineering and therefore cannot give relevant guidance. Further theoretical study is needed.

In this paper, a two-dimensional plane drainage model is proposed for vacuum preloading combined with the electro-osmosis method considering both anode and cathode flows. New EKG materials are used as electrodes in the model. Considering the smearing effect, the analytical solution is derived to describe the behavior of the pore water pressure and the consolidation process around the anode. The laboratory experiment is developed to obtain a measured value to verify the proposed analytical solution.

## Basic assumptions and governing equations

### Analytical model and basic assumptions

**Fig 1:**
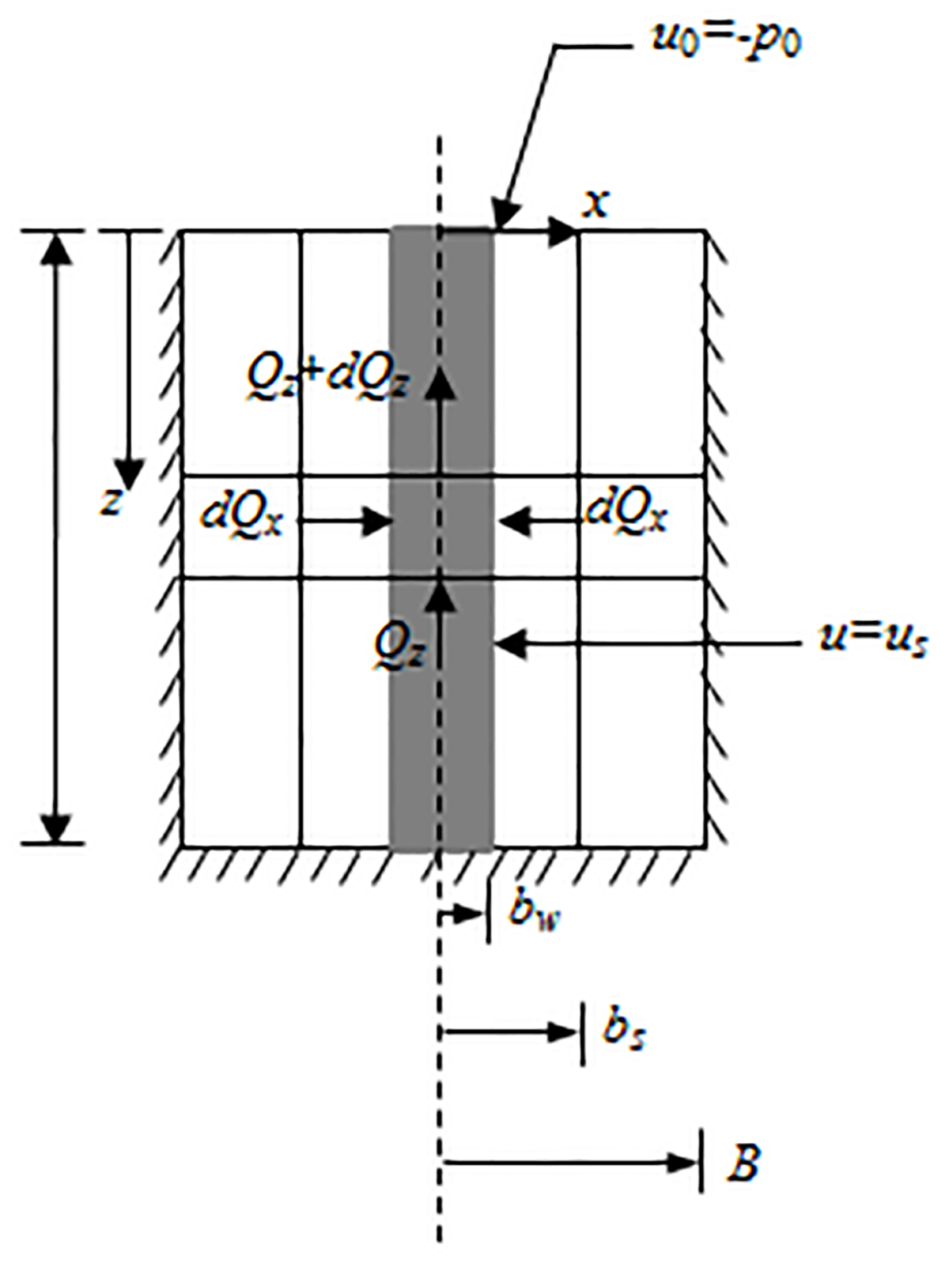
Analytical model. Figure 1 illustrated the unit cell adopted for plane strain conditions. The change of flow occurred in the *z* direction and *x* direction. (A) *dQ*_x_ demonstrated the flow in the *x* direction of the drain from the entrance to the exit of the unit cell. (B) The total change in flow from the entrance face to the exit face of the unit cell was given by *Q*_z_. (C) Considering the incompressibility of water, *dQ*_x_ was equal to–*dQ*_z_. The equation can be established according to plane strain unit cell.

Aiming at electrodes arranged in the shape of a rectangle in the engineering field, a vertical cross section of the unit cell, which shows the flow condition in the vertical drain with a central EKG in the anode is shown in Fig 1. In other words, a basic unit of soil consists of one anode and one cathode.

A rectangular coordinate system is established in the analytical model. The drain board is simplified as the shape of a rectangle with a width of 2*b*_w_. The potential between the anode and cathode is *U*, and it is provided by the DC electrical source. Negative pressure *u*_0_ = -*p*_0_ is applied on the surface of the soil due to the effect of vacuum preloading. *k*_h_ is radial hydraulic conductivity, and *k*_e_ is the electro-osmosis conductivity.

**Fig 2:**
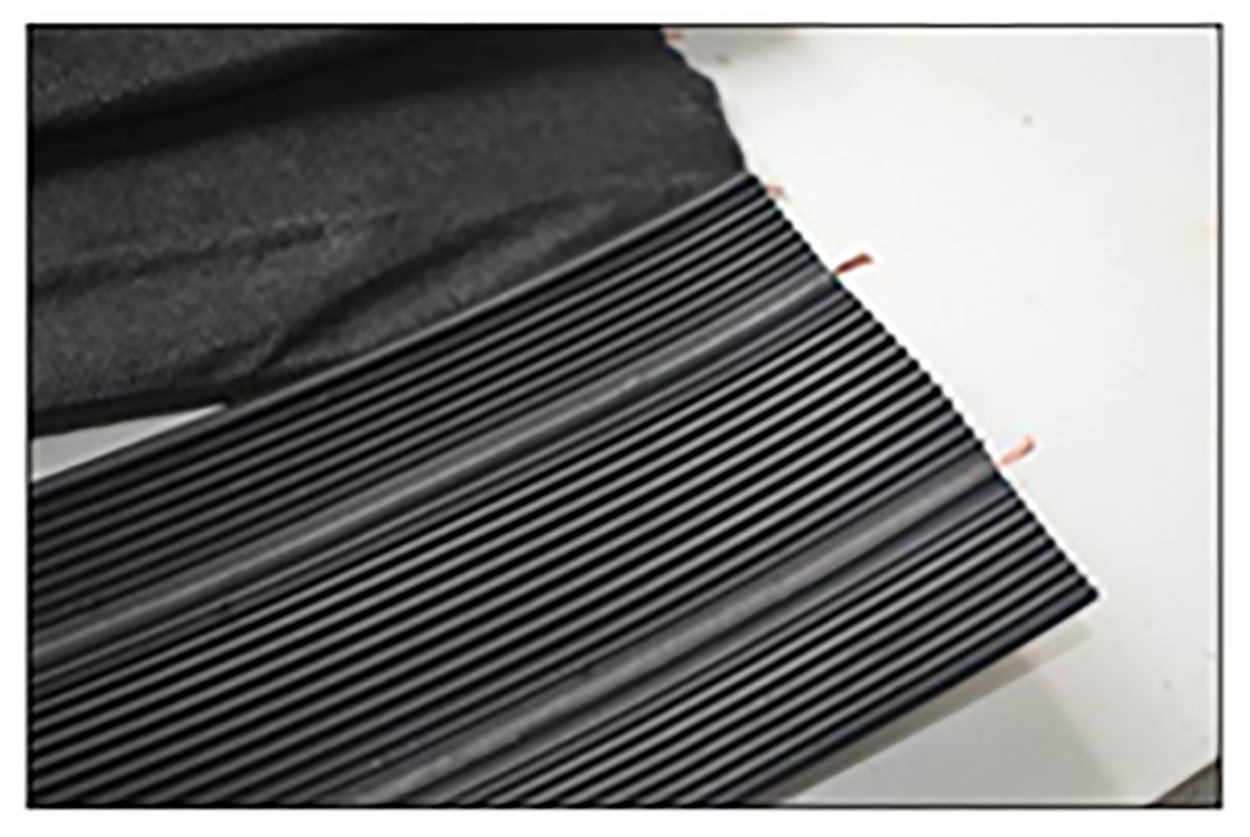
Real product of the EKG electrode. The functions of EKG materials included drainage, electric conduction and corrosion resistance. This new material can relatively shorten the consolidation time when compared with the conventional test and can also avoid the electrolytic reaction of metal electrodes during the test. Thus, the EKG can make the coupling between the electric seepage field and the hydraulic seepage field more effectively.

**Fig 3:**
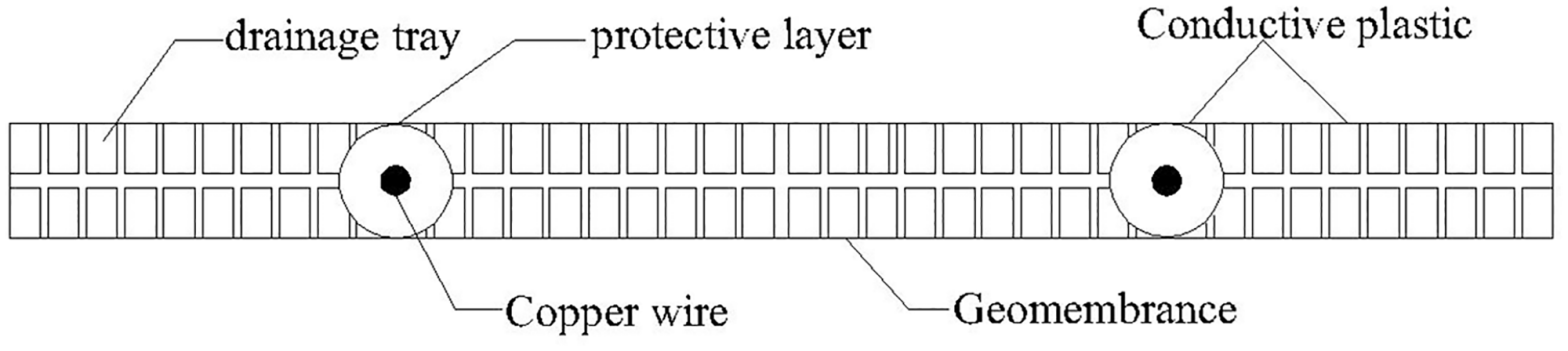
Diagram for the EKG electrode. Both sides of the EKG were covered with drainage tray, copper wires and a conductive geomembrane. It contained polyethylene, carbon black, and graphite (mass ratio = 1: (0.3~0.35): (0.05~0.1)). The copper wires were protected by the electric polymer plastics and transmission electric energy during the test. So, the mental corrosion can be avoided to guarantee the effect of experiment.

Different from conventional vacuum preloading combined with the electro-osmosis method. In this paper, a new type of corrosion resistant electrode material, named EKG, is used. The real EKG product can be seen in Fig 2, and its structure can be seen in Fig 3. The functions of EKG materials included drainage, electric conduction and corrosion resistance. Both sides of the EKG were covered with a groove for drainage, brass wires and a conductive geomembrane. It contained polyethylene, carbon black, and graphite (mass ratio = 1: (0.3~0.35): (0.05~0.1)). The brass wires were protected by the electric polymer plastics and transmission electric energy during the test. So, the mental corrosion can be avoided to guarantee the effect of experiment. This new material can relatively shorten the consolidation time when compared with the conventional test and can also avoid the electrolytic reaction of metal electrodes during the test. Thus, EKG can make the coupling between the electric seepage field and the hydraulic seepage field more effective. An area of influence called the smear zone was formed with a width of 2*b*_s_. A central EKG in the anode was under the effect of the provided power. The radial hydraulic conductivity was less than *k*_h_. Furthermore, an area of influence with the width of 2*b*_c_ and a central EKG was also formed under the effect of the vertical drain board. Specially, the two-dimensional plane model showed in Fig 1 was just a vertical cross section of a unit cell. The experimental model was simplified to show the flow condition in the vertical drain. Therefore, 2*b*_c_ was not equal to the space between electrodes.

Basic assumptions in this paper are as follows:

1. Laminar flow through the soil (Darcy’s law) is adopted. Flow is not permitted at the outer boundary of the unit cell. Only radial flow is permitted for relatively long vertical drains. The flow caused by the electrical gradient can be overlaid with flow caused by the hydraulic gradient.
2. The soil is fully saturated and homogeneous. The decrement of soil particles and pore-water during the process of consolidation can be neglected. The drainage amount discharged from the unit cell of soil is equal to the decrement of soil volume.
3. Based on the equal-strain concept, all vertical strains at any given depth *z* are assumed to be equal. Compressive strains are allowed to occur in the vertical direction only. The permeability of the soil is assumed to be constant during consolidation.
4. The decrease in vacuum pressure along the vertical of the drainage plate should be considered. The vacuum pressure on the surface of the soil is assumed to be–*p*_0_, and the vacuum pressure at the bottom of the drainage plate is–*k*_1_*p*_0_; the vacuum pressure is -*p*_0_(1-(1-*k*_1_))*z*/*l* at any other given depth *z*; *k*_1_ is the vacuum transfer coefficient, 0<*k*_1_<1. The effect of vacuum preloading produces an area of influence with a central EKG, and the width of the influence area is 2*b*_c_.

### Brief equation derivation

The pore-water flow velocity of vacuum preloading combined with electro-osmosis is coupled with hydraulic gradient flow and electrical gradient flow according to assumption Eq (1):

$$\text{Anode: }v=k_{h}i_{h}-k_{e}i_{e}$$ (1)

$$\text{Cathode: }v=k_{h}i_{h}+k_{e}i_{e}$$ (2)

where *v* is the pore-water flow velocity; *k*_h_ and *k*_e_ are the radial hydraulic conductivity and electro-osmosis conductivity, respectively; and *i*_h_ and *i*_e_ are the hydraulic gradient and electrical gradient, respectively.

*i*_e_ can be calculated according to assumption Eq (6):

$$i_{\text{e}}=grand\left(\varphi \right)=U_{0}/2B$$ (3)

where 2*B* is the space between electrodes, and *U*_0_ is the effective voltage.

### Governing equations

The analytical solution in the anode is derived as an example. Water flow to the anode is the difference of the electrical gradient and the hydraulic gradient:

$$\frac{\partial Q}{\partial t}=\left(\frac{k_{h,ps}}{\gamma_{w}}\frac{\partial u}{\partial x}-k_{e}i_{e}\right)2ldz=\left(\frac{k_{h,ps}}{\gamma_{w}}\frac{\partial u}{\partial x}-k_{e}\frac{U_{0}}{2B}\right)2ldz$$ (4)

where *Q* is the water flow through soil, *u* is the excess pore water pressure caused by heap loading and electro-osmosis, *γ*_w_ is the specific weight of water and *k*_h,ps_ and *k*_e_ are the radial conductivity and electro-osmosis conductivity, respectively; and subscript “ps” denotes the plane strain condition.

The drainage amount discharged from the unit cell of soil is equal to the decrement of soil volume according to assumption Eq (2), and the rate of volume change is:

$$\frac{\partial V}{\partial t}=\frac{\partial \varepsilon }{\partial t}\left(b_{c}-x\right)ldz$$ (5)

Furthermore, the equation described as $\frac{\partial Q}{\partial t}=\frac{\partial V}{\partial t}$ can be derived due to the water flowing to the vertical drains and is equal to the amount of drainage:

$$\left(\frac{k_{h,ps}}{\gamma_{w}}\frac{\partial u}{\partial x}-k_{e}\frac{U_{0}}{2B}\right)2ldz=\frac{\partial \varepsilon }{\partial t}\left(b_{c}-x\right)ldz$$ (6)

Based on Eq (6), the excess pore water pressure gradient outside the smear zone is:

$$\frac{\partial u}{\partial x}=\frac{\gamma_{w}}{2k_{h,ps}}\left(\frac{\partial \varepsilon }{\partial t}\left(b_{c}-x\right)+k_{e}\frac{\gamma_{w}}{k_{h,ps}}\frac{U_{0}}{B}\right)$$ (7)

where *u* is the pore-water pressure in the undisturbed zone.

Because of the change in radial conductivity only in the smear zone and outside, the excess pore water pressure gradient in the smear zone is:

$$\frac{\partial u_{s}}{\partial x}=\frac{\gamma_{w}}{2k_{s,ps}}\left(\frac{\partial \varepsilon }{\partial t}\left(b_{c}-x\right)+k_{e}\frac{\gamma_{w}}{k_{s,ps}}\frac{U_{0}}{B}\right)$$ (8)

where *u*_s_ is the pore-water pressure in the smear zone, and *k*_s,ps_ is the permeability in the smear zone.

The equation of pore-water pressure changing with time along the vertical direction can be established based on Eq (8). As shown in Fig 1, the real width of the drain is 2*b*_w_ at a certain time *t*, and a cuboid slice with unit width is taken out from the drain, the height of which is assumed to be *dz*.

$$dQ_{z}=\frac{q_{w,ps}}{\gamma_{w}}\frac{\partial^{2}u}{\partial z^{2}}dzdt$$ (9)

where *q*_w,ps_ is the well discharge capacity in the smear zone for *x*≤*b*_w_.

At the drain boundary, the change of flow from the entrance face to the exit face of the slice in a one-sided drain is given by:

$$dQ_{x}=\frac{lk_{s,ps}}{\gamma_{w}}\frac{\partial u_{s}}{\partial x}dzdt$$ (10)

As the water is assumed to be incompressible according to assumption Eq (2), the following equation should be satisfied:

$$dQ_{z}+2dQ_{x}=0$$ (11)

Furthermore, at the drain boundary, it is assumed that a sudden drop in pore pressure does not occur; hence, *u* = *u*_s_ substituting Eqs (9) and (10) into Eq (12):

$${\left(\frac{q_{w,ps}}{\gamma_{w}}\frac{\partial^{2}u}{\partial z^{2}}\right)}_{x=b_{w}}+2{\left(\frac{lk_{s,ps}}{\gamma_{w}}\frac{\partial u}{\partial x}\right)}_{x=b_{w}}=0$$ (12)

Substituting Eq (8) into Eq (12) at the boundary, the quadratic function of pore-water pressure along the vertical direction can be given by:

$$\frac{\partial^{2}u_{s}}{\partial z^{2}}=-\frac{\gamma_{w}}{q_{w,ps}}\frac{\partial \varepsilon }{\partial t}\left(b_{c}-b_{w}\right)-\frac{\gamma_{w}}{q_{w,ps}}\frac{U_{0}}{B}$$ (13)

### Boundary conditions and transfusion continuity condition

1. Boundary conditions:
  - *z* = 0, *u*_s_ = -*p*_0;_
  - *z* = *l*, $\frac{\partial u_{s}}{\partial z}=p_{0}\left(1-k_{1}\right)/l$;
2. Transfusion continuity condition:

$$\frac{\partial \varepsilon }{\partial t}=-m_{v}\frac{\partial u}{\partial t}$$

## Equation solutions

By integrating the Eq (13) over the *z* variable,: the following equation can be obtained:

$$\frac{\partial u_{s}}{\partial z}=-\frac{2\gamma_{w}}{q_{w,ps}}z\left[\frac{\partial \varepsilon }{\partial t}\left(b_{c}-b_{w}\right)+k_{e}\frac{U_{0}}{2b_{c}}\right]+C_{1}$$ (14)

The pore-water pressure at *x* = *bw* is determined from Eq (14):

$${\left(u_{s}\right)}_{x=b_{w}}=-\frac{\gamma_{w}}{q_{w,ps}}z^{2}\left(\frac{\partial \varepsilon }{\partial t}\left(b_{c}-b_{w}\right)+k_{e}\frac{U_{0}}{B}\right)+C_{1}z+C_{2}$$ (15)

Subject the boundary conditions at *z* = 0, *u*_s_ = -*p*_0_, *z* = *l*, $\frac{\partial u_{s}}{\partial z}=p_{0}\left(1-k_{1}\right)/l$ to Eq (15), and *C*_1_ and *C*_2_ can be determined:

$$\begin{array}{l} C_{1}=\frac{p_{0}\left(1-k_{1}\right)}{l}+\frac{\gamma_{w}l}{q_{w,ps}}\left(\frac{\partial \varepsilon }{\partial t}\left(b_{c}-b_{w}\right)+k_{e}\frac{U_{0}}{B}\right)\\ C_{2}=-p_{0} \end{array}$$

The excess pore water pressure at *x* = *b*_w_ can be determined by:

$${\left(u_{s}\right)}_{x=b_{w}}=-\frac{\gamma_{w}}{q_{w,ps}}z^{2}\left(\frac{\partial \varepsilon }{\partial t}\left(b_{c}-b_{w}\right)+k_{e}\frac{U_{0}}{B}\right)+\left\{\frac{p_{0}\left(1-k_{1}\right)}{l}+\frac{\gamma_{w}l}{q_{w,ps}}\left(b_{c}-b_{w}\right)+k_{e}\frac{U_{0}}{B}\right\}z-p_{0}$$ (16)

The pore-water pressure in the smear zone can be determined by integrating Eq (8) in the *z* direction subject to the *u*_s_ given in Eq (16). By assuming *u*_s_ = (*u*_s_)_*x* = *b*w_ (see Eq (16)) at the internal interface *x* = *b*_w_, the pore-water pressure in the smear zone *u*_s_ can be expressed by:

$$\begin{array}{l} u_{s}=-\frac{\gamma_{w}}{2k_{s,ps}}\frac{\partial \varepsilon }{\partial t}x^{2}+\frac{\gamma_{w}}{2k_{s,ps}}\left(\frac{\partial \varepsilon }{\partial t}b_{c}+k_{e}\frac{U_{0}}{B}\right)x-\frac{\gamma_{w}}{q_{w,ps}}\left(b_{c}-b_{w}+k_{e}\frac{U_{0}}{B}\right)z^{2}+\{\frac{p_{0}\left(1-k_{1}\right)}{l}+\\ \frac{\gamma_{w}l}{q_{w,ps}}\left(\frac{\partial \varepsilon }{\partial t}\left(b_{c}-b_{w}\right)+k_{e}\frac{U_{0}}{B}\right)\}z+[\frac{\gamma_{w}}{2k_{s,ps}}\frac{\partial \varepsilon }{\partial t}b_{w}^{2}-\frac{\gamma_{w}}{2k_{s,ps}}\left(\frac{\partial \varepsilon }{\partial t}b_{c}+k_{e}\frac{U_{0}}{B}\right)b_{w}-p_{0}] \end{array}$$ (17)

for *b*_w_≤*x*≤*b*_s_.

At the boundary of the smear zone *x* = *b*_s_, pore-water pressure is permanent, and it can be described as $u_{x=b_{s}}={\left(u_{s}\right)}_{x=b_{s}}$. The governing Eq (7) can be written as:

$$\begin{array}{l} u=-\frac{\gamma_{w}}{2k_{h,ps}}\frac{\partial \varepsilon }{\partial t}x^{2}+\frac{\gamma_{w}}{2k_{h,ps}}\left(\frac{\partial \varepsilon }{\partial t}b_{c}+k_{e}\frac{U_{0}}{2B}\right)x-\frac{\gamma_{w}}{2k_{s,ps}}\frac{\partial \varepsilon }{\partial t}b_{s}^{2}+\frac{\gamma_{w}}{2k_{s,ps}}\left(\frac{\partial \varepsilon }{\partial t}b_{c}+k_{e}\frac{U_{0}}{2B}\right)b_{s}-\\ \frac{\gamma_{w}}{q_{w,ps}}\left(\frac{\partial \varepsilon }{\partial t}\left(b_{c}-b_{w}\right)+k_{e}\frac{U_{0}}{B}\right)z^{2}+\{\frac{p_{0}\left(1-k_{1}\right)}{l}+\frac{\gamma_{w}l}{q_{w,ps}}\left(\frac{\partial \varepsilon }{\partial t}\left(b_{c}-b_{w}\right)+k_{e}\frac{U_{0}}{B}\right)\}z+[\frac{\gamma_{w}}{2k_{s,ps}}\frac{\partial \varepsilon }{\partial t}\\ b_{w}^{2}-\frac{\gamma_{w}}{2k_{s,ps}}\left(\frac{\partial \varepsilon }{\partial t}b_{c}+k_{e}\frac{U_{0}}{2B}\right)b_{w}-p_{0}]-\frac{\gamma_{w}}{k_{h,ps}}(\frac{\partial \varepsilon }{\partial t}b_{c}+k_{e}\frac{U_{0}}{B})b_{s}+\frac{\gamma_{w}}{2k_{h,ps}}\frac{\partial \varepsilon }{\partial t}b_{s}^{2} \end{array}$$ (18)

for *b*_s_≤*x*≤*b*_c_.

The mean pore-water pressure ($\bar{u}$) in the *z* direction can be derived as:

$$\bar{u}=\frac{\int_{b_{w}}^{b_{s}}u_{s}dx+\int_{b_{s}}^{b_{c}}udx}{\left(b_{c}-b_{w}\right)}$$ (19)

Substituting Eqs (17) and (18) into Eq (19), the pore-water pressure can be given by:

$$\begin{array}{l} \bar{u}=-\frac{\gamma_{w}}{6\left(b_{c}-b_{w}\right)}\frac{\partial \varepsilon }{\partial t}\left(\frac{\left(b_{s}^{3}-b_{w}^{3}\right)}{2k_{s,ps}}+\frac{\left(b_{c}{}^{3}-b_{s}^{3}\right)}{2k_{h,ps}}\right)+\frac{\gamma_{w}}{2\left(b_{c}-b_{w}\right)}\left(\frac{\partial \varepsilon }{\partial t}b_{c}+k_{e}\frac{U_{0}}{B}\right)[\frac{\left(b_{s}^{2}-b_{w}^{2}\right)}{2k_{s,ps}}+\\ \frac{\left(b_{c}{}^{2}-b_{s}^{2}\right)}{2k_{h,ps}}]+\frac{\gamma_{w}b_{s}\left(b_{c}-b_{s}\right)}{b_{c}-b_{w}}\left(\frac{\partial \varepsilon }{\partial t}b_{c}+k_{e}\frac{U_{0}}{B}\right)\left(\frac{1}{2k_{s,ps}}-\frac{1}{2k_{h,ps}}\right)-\frac{\gamma_{w}b_{s}^{2}}{2\left(b_{c}-b_{w}\right)}\frac{\partial \varepsilon }{\partial t}\left(\frac{1}{2k_{s,ps}}-\frac{1}{2k_{h,ps}}\right)\\ -\frac{\gamma_{w}}{q_{w,ps}}\left[\frac{\partial \varepsilon }{\partial t}\left(b_{c}-b_{w}\right)+k_{e}\frac{U_{0}}{B}\right]z^{2}+\{\frac{p_{0}\left(1-k_{1}\right)}{l}+\frac{2\gamma_{w}l}{q_{w,ps}}\left[\frac{\partial \varepsilon }{\partial t}\left(b_{c}-b_{w}\right)+k_{e}\frac{U_{0}}{B}\right]\}z+[\frac{\gamma_{w}}{2k_{s,ps}}\frac{\partial \varepsilon }{\partial t}b_{w}^{2}\\ -\frac{\gamma_{w}}{2k_{s,ps}}\left(\frac{\partial \varepsilon }{\partial t}b_{c}+k_{e}\frac{U_{o}}{B}\right)b_{w}-p_{0}] \end{array}$$ (20)

Combining Eq (20) with the well-known compressibility relationship $\frac{\partial \varepsilon }{\partial t}=-m_{v}\frac{\partial u}{\partial t}$ gives:

$$\begin{array}{l} \bar{u}=m_{v}\{\frac{\gamma_{w}}{6\left(b_{c}-b_{w}\right)}\left(\frac{\left(b_{s}^{3}-b_{w}^{3}\right)}{2k_{s,ps}}+\frac{\left(b_{c}{}^{3}-b_{s}^{3}\right)}{2k_{h,ps}}\right)-\frac{\gamma_{w}}{2\left(b_{c}-b_{w}\right)}\left[\frac{\left(b_{s}^{3}-b_{w}^{3}\right)}{2k_{s,ps}}+\frac{\left(b_{c}{}^{3}-b_{s}^{3}\right)}{2k_{h,ps}}\right]-\frac{\gamma_{w}b_{s}}{\left(b_{c}-b_{w}\right)}\\ \left(b_{c}-b_{s}\right)b_{c}\left(\frac{1}{2k_{s,ps}}-\frac{1}{2k_{h,ps}}\right)+\frac{\gamma_{w}b_{s}^{2}\left(b_{c}-b_{s}\right)}{2\left(b_{c}-b_{w}\right)}\left(\frac{1}{2k_{s,ps}}-\frac{1}{2k_{h,ps}}\right)+\frac{\gamma_{w}z^{2}}{q_{w,ps}}\left(b_{c}-b_{w}\right)-\frac{\gamma_{w}lz}{q_{w,ps}}\left(b_{c}-b_{w}\right)\\ -\frac{\gamma_{w}b_{w}^{2}}{2k_{s,ps}}+\frac{\gamma_{w}b_{c}b_{w}}{2k_{s,ps}}\}\frac{\partial \overset{-}{u}}{\partial t}+\{\frac{k_{e}U_{0}\gamma_{w}}{2B\left(b_{c}-b_{w}\right)}[\frac{\left(b_{s}^{2}-b_{w}^{2}\right)}{2k_{s,ps}}+\frac{\left(b_{c}{}^{2}-b_{s}^{2}\right)}{2k_{h,ps}}]+\frac{k_{e}U_{0}\gamma_{w}b_{s}\left(b_{c}-b_{s}\right)}{B\left(b_{c}-b_{w}\right)}\left(\frac{1}{2k_{s,ps}}-\frac{1}{2k_{h,ps}}\right)\\ -k_{e}\frac{U_{0}}{B}\frac{\gamma_{w}}{q_{w,ps}}z^{2}+\frac{p_{0}\left(1-k_{1}\right)z}{l}+\frac{\gamma_{w}l}{q_{w,ps}}\frac{k_{e}U_{0}z}{B}-\frac{\gamma_{w}}{2k_{s,ps}}k_{e}\frac{U_{0}}{B}b_{w}-p_{0}\} \end{array}$$ (21)

Eq (21) can be simplified to $\bar{u}=\frac{\partial \bar{u}}{\partial t}M+N$, where:

$$\begin{array}{l} M=m_{v}\{\frac{\gamma_{w}}{6\left(b_{c}-b_{w}\right)}\left(\frac{\left(b_{s}^{3}-b_{w}^{3}\right)}{2k_{s,ps}}+\frac{\left(b_{c}{}^{3}-b_{s}^{3}\right)}{2k_{h,ps}}\right)-\frac{\gamma_{w}b_{c}}{2\left(b_{c}-b_{w}\right)}\left[\frac{\left(b_{s}^{3}-b_{w}^{3}\right)}{2k_{s,ps}}+\frac{\left(b_{c}{}^{3}-b_{s}^{3}\right)}{2k_{h,ps}}\right]-\frac{\gamma_{w}b_{s}}{\left(b_{c}-b_{w}\right)}\\ \left(b_{c}-b_{s}\right)b_{c}\left(\frac{1}{2k_{s,ps}}-\frac{1}{2k_{h,ps}}\right)+\frac{\gamma_{w}b_{s}^{2}\left(b_{c}-b_{s}\right)}{2\left(b_{c}-b_{w}\right)}\left(\frac{1}{2k_{s,ps}}-\frac{1}{2k_{h,ps}}\right)+\frac{\gamma_{w}z^{2}}{q_{w,ps}}\left(b_{c}-b_{w}\right)-\frac{\gamma_{w}lz}{q_{w,ps}}\left(b_{c}-b_{w}\right)\\ -\frac{\gamma_{w}b_{w}^{2}}{2k_{s,ps}}+\frac{\gamma_{w}b_{c}b_{w}}{2k_{s,ps}}\} \end{array}$$ (22)

$$\begin{array}{l} N=\{\frac{k_{e}U_{0}\gamma_{w}}{2B\left(b_{c}-b_{w}\right)}\left[\frac{\left(b_{s}^{2}-b_{w}^{2}\right)}{2k_{s,ps}}+\frac{\left(b_{c}{}^{2}-b_{s}^{2}\right)}{2k_{h,ps}}\right]+\frac{k_{e}U_{0}\gamma_{w}b_{s}\left(b_{c}-b_{s}\right)}{B\left(b_{c}-b_{w}\right)}\left(\frac{1}{2k_{s,ps}}-\frac{1}{2k_{h,ps}}\right)-k_{e}\frac{U_{0}}{B}\\ \frac{\gamma_{w}}{q_{w,ps}}z^{3}+\frac{p_{0}\left(1-k_{1}\right)z}{l}+\frac{\gamma_{w}l}{q_{w,ps}}\frac{k_{e}U_{0}z^{2}}{B}-\frac{\gamma_{w}}{2k_{s,ps}}k_{e}\frac{U_{0}}{B}b_{w}-p_{0}\} \end{array}$$ (23)

Substituting the initial condition *t* = 0, $\bar{u}=0$ into equation $\bar{u}=M\frac{\partial \bar{u}}{\partial t}+N$ can be given by:

$$\bar{u}=e^{\frac{t}{M}}\cdot e^{\text{ln}\left(-N\right)}+N$$ (24)

Eq (24) demonstrates the mean pore-water pressure at any given depth *z*.

The average degree of consolidation in the *z* direction can now be evaluated conveniently by the following equation:

$$U_{r}=1-\frac{\bar{u}-{\bar{u}}_{f}}{u_{0}-{\bar{u}}_{f}}$$ (25)

where *u*_0_ is the initial pore water pressure; ${\bar{u}}_{f}$ is the pore-water pressure at the end of foundation treatment. The average degree of consolidation in the anode can be given by substituting Eq (24) into Eq (25).

## Analysis of example

### Soil sample

The basic parameters for vacuum preloading combined with electro-osmotic consolidation in the laboratory experiment are shown in Table 1.

**Table 1:** Basic parameters for the laboratory experiment.

Parameter	Value	Parameter	Value	Parameter	Value	Parameter	Value
Water content *w*_0_(%)	50	Plastic index *I*_p_	18.3	Electro-osmotic conductivity *k*_e_ (cm^2^/sv)	5×10^−5^	Value of vacuum preloading *p*_0_ (kPa)	60
Proportion *G*_s_	2.75	Clay content (%)	78.6	Width of the EKG drain *b*_w_ (mm)	6.5	Well discharge capacity of EKG *q*_w,ps_ (m^3^/s)	18
Liquid limit *w*_L_ (%)	42.5	Hydraulic conductivity conductivity (cm/s)	4.5×10^−6^	Width of the smear zone for EKG *b*_s_ (mm)	110	Depth of the model *l* (mm)	400
Plastic limit *w*_p_ (%)	24.2	Hydraulic conductivity of the smear zone *k*_s,ps_ (cm/s)	1.19×10^−6^	Width of the model *b*_c_ (mm)	100	Depth of the piezometer *z* (mm)	200

### Experiment apparatus

**Fig 4:**
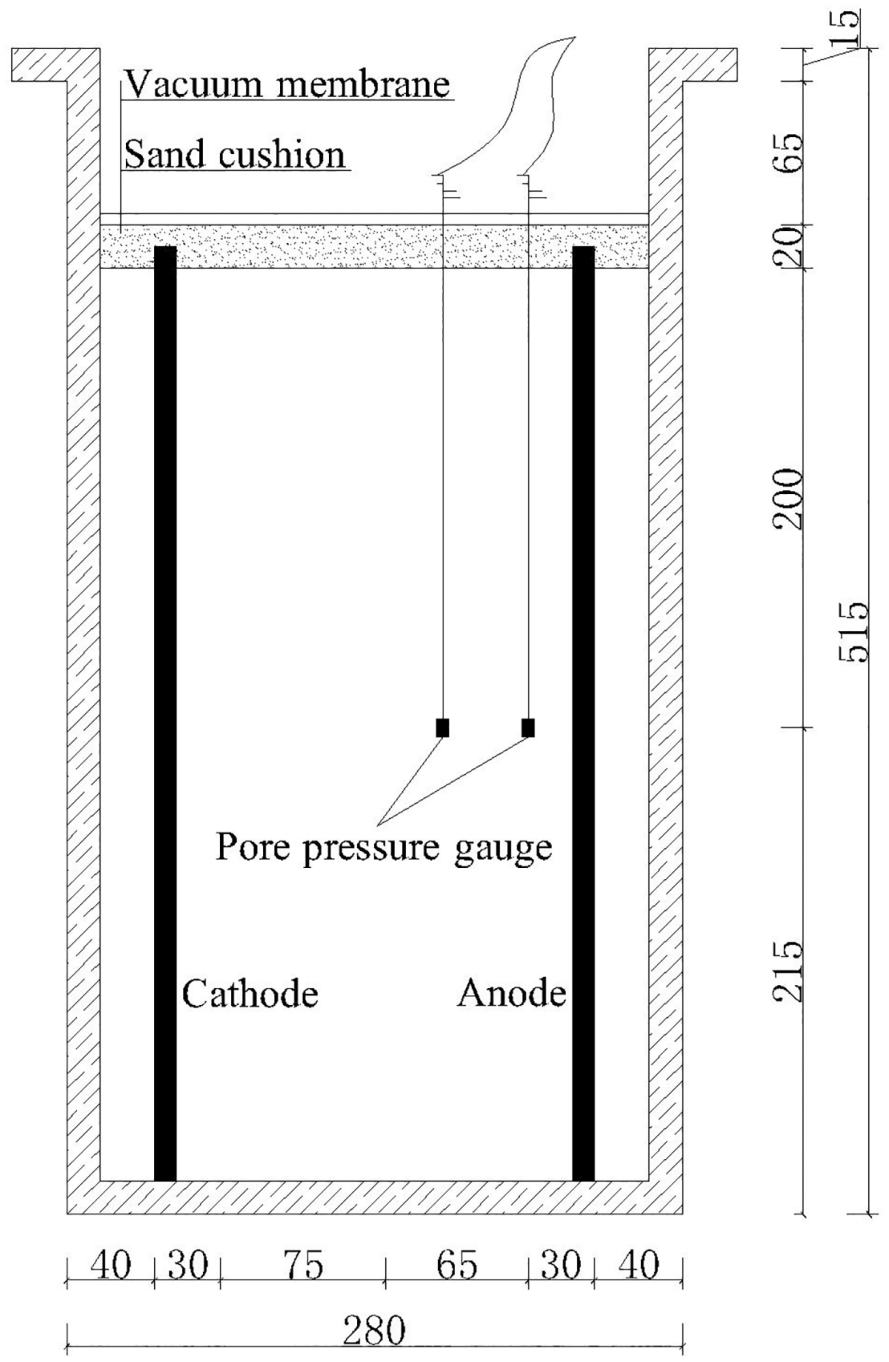
Diagram of model device. The model device was made of organic glass (L×W×D = 250 mm×200 mm×500 mm). It contained EKG electrodes, pore pressure gauge, vacuum membrane and sand cushion. The sand cushion was placed at the top of the soil sample. A vacuum membrane was used to isolate the sample from the air.

**Fig 5:**
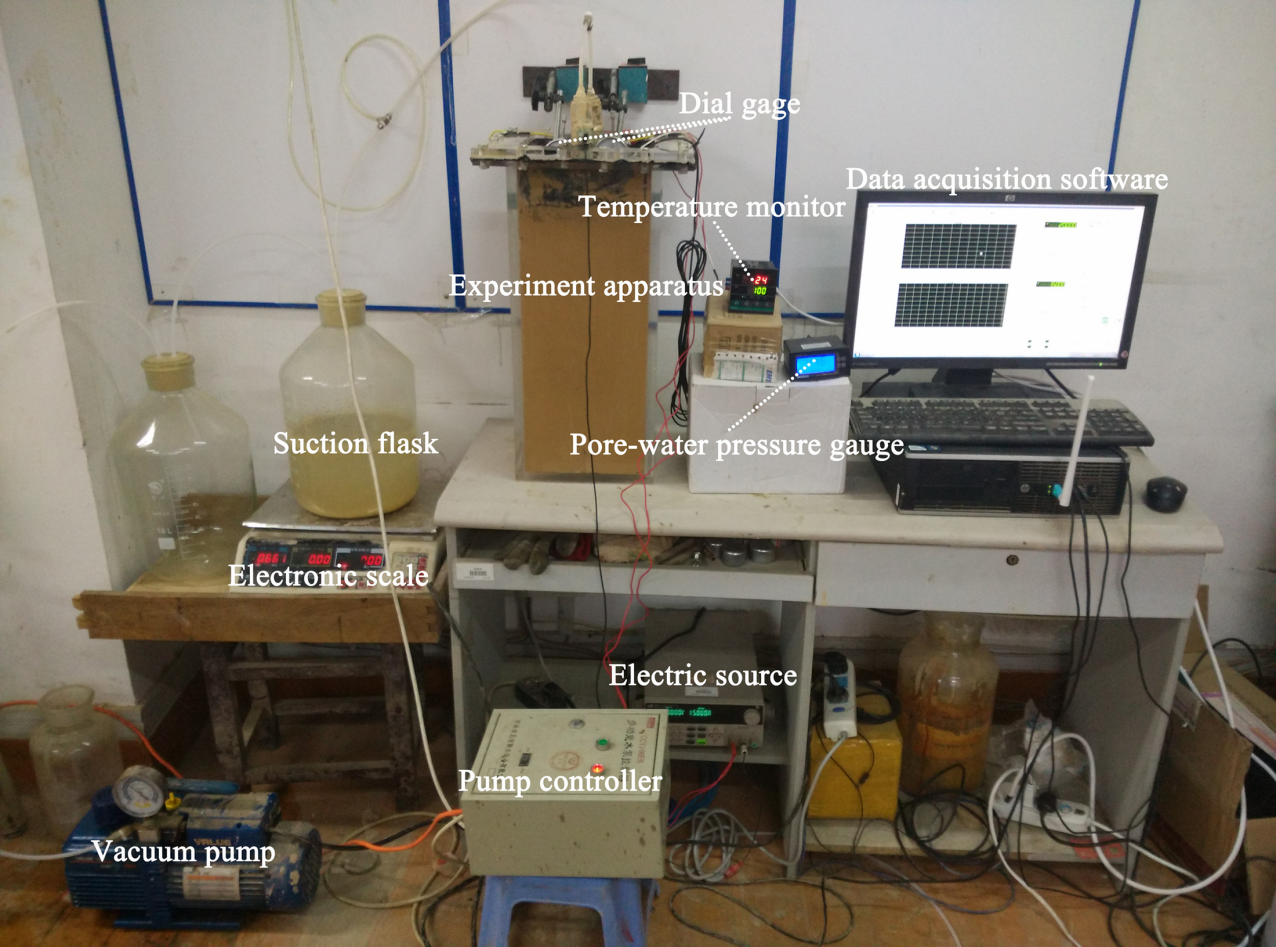
Real photo of experiment apparatus. The panorama of test apparatus was showed. (A) Model device expressed the process of test. The EKG electrodes were linked to the electric source through electric wires. (B) The pore pressure gauges were linked to the readout instrument through electric wires to record the data. (C) Vacuum pump and pump controller were used to control the vacuum degree.

As shown in Fig 4, vacuum preloading combined with the electro-osmotic method was investigated by using EKG in a self-developed apparatus. The real photo of experiment apparatus can be seen in Fig 5. The model device was made of organic glass (L×W×D = 250 mm×200 mm×500 mm). The rated power of the vacuum pump was 750 W, and the pumping rate was 14.4 m^3^/h. A DC power supply was used in the experiment with an output voltage of 20 V and a maximum output power of 60 W.

Epoxy resin smearing was carried out for lubrication before the experiment. EKG materials used as electrodes were fixed at the bottom of the model, which was linked to a connector used as a bridge for the connection between the EKG electrode and drain-pipe. The length of the EKG electrode was 420 mm. The soil sample thickness was 400 mm. To make the soil sample denser, it was divided into four layers. Each layer was filled and carefully compacted. As shown in Fig 4, there were two pore pressure gauges that were 20 mm and 75 mm away from the anode and dipped at a depth of 200 mm. Disturbing the pore pressure gauges in the seepage field can be neglected due to their small size. The pore pressure gauges were linked to the readout instrument through electric wires to record the data.

A sand cushion was placed at the top of the soil sample when the electrodes and pore pressure gauges were embedded in the soil. Then, a vacuum membrane was used to isolate the sample from the air. The EKG electrodes were linked to the electric source through electric wires. Meanwhile, a drain-pipe connected with pneumatic tube was linked to a vacuum saturation cylinder. Finally, the examination was carried out to guarantee the airtightness of the whole apparatus.

### Results and discussion

**Fig 6:**
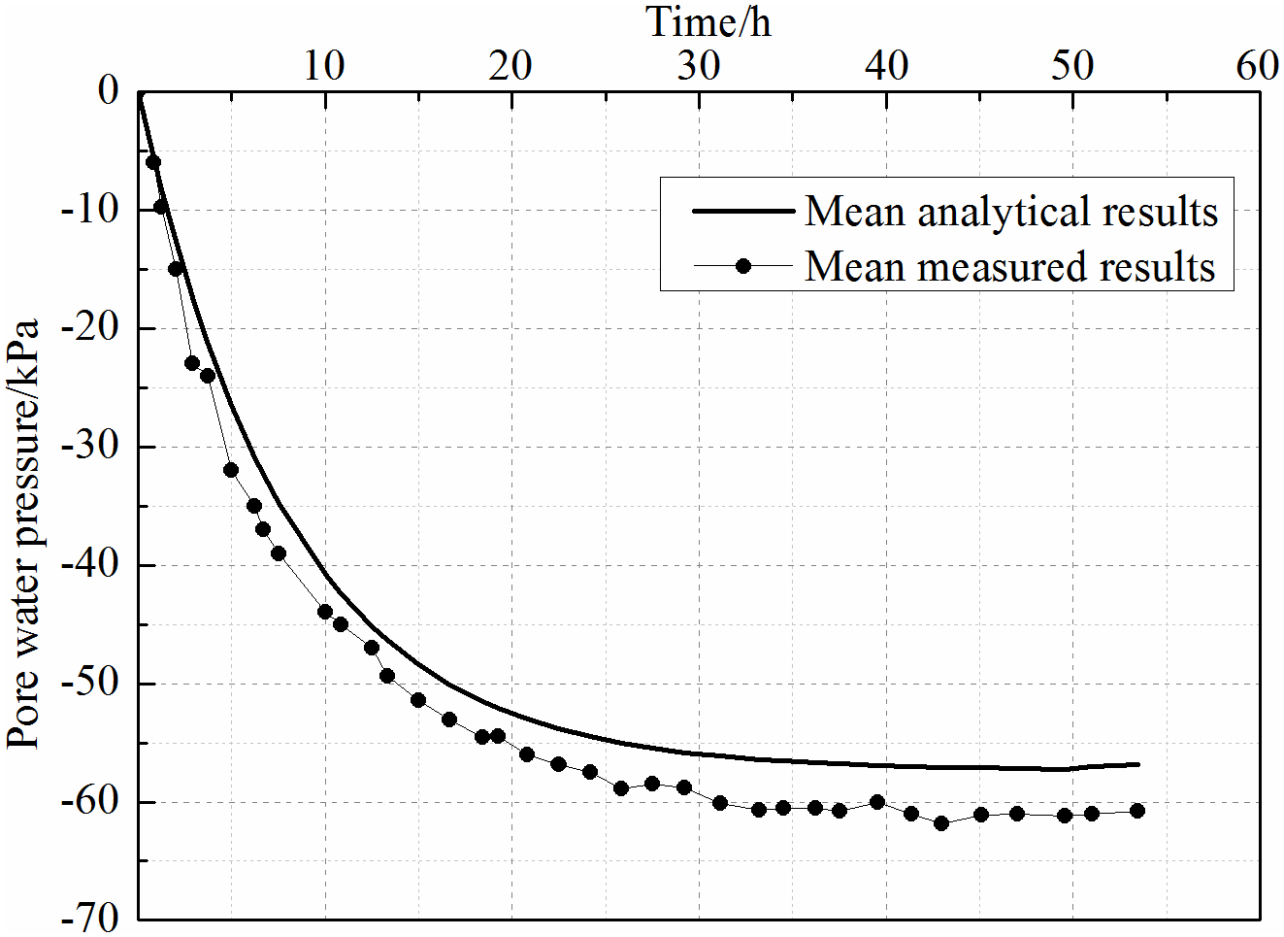
Comparison of analytical result and measures result. The trends of the mean measured value and the mean analytical value processes were comparable. The mean analytical result agreed well with the mean measured value during the first 5 h. Different value appeared 5 h later, when the mean measured result was larger than the mean analytical result. Electrolytic exothermic and chemical reactions among ions can give rise to changes in the pore-water pressure, which was neglected by the analytical derivation The final mean measured value was about 61 kPa.

Pore-water pressure in the anode was collected through data acquisition software during the experiment. Mean analytical results were calculated by substituting the parameters listed in Table 1 into Eq (21). The exact value of *k*_1_ in experiment is 0.85. The comparison of mean measured results and average analytical results are demonstrated in Fig 6. The average value of the two measured points was calculated as the mean measured result in the interlayer of the anode. Specially, based on the equal-strain concept, all vertical strains at any given depth *z* were assumed to be equal. The dissipation of pore-water pressure was affected by the joint effect between the vacuum preloading and electro-osmosis. The average degree of consolidation in different position only can be derived from the same average pore-water pressure at any given depth *z*. So, the mean pore-water pressure should be studied particularly. Fig 6 shows that the trends of the mean measured value and the mean analytical value processes are comparable. The corrosion resistance and siltation resistance of EKG electrodes is considered to ensure experiment process stability. Specifically, the mean measured value in the late period of the experiment is more accurate when compared with a conventional metal electrode. The mean measured value using mental electrodes is about 51 kPa. Due to the stacking effect of vacuum preloading and electro-osmosis, the final mean measured value is about 61 kPa, it is greater than 60 kPa. In other words, the vacuum preloading and electro-osmosis can both accelerate the dissipation of pore-water pressure.

As shown in Fig 6, the mean analytical result agrees well with the mean measured value during the first 5 h. Different value appears 5 h later, when the mean measured result is larger than the mean analytical result. Electrolytic exothermic and chemical reactions among ions can give rise to changes in the pore-water pressure [16], which was neglected by the analytical derivation. Thus, the mean analytical value is smaller than the mean measured value. Furthermore, changes in the temperature field around the anode can also promote pore-water pressure dissipation. In addition, considering the equal-strain concept, all vertical strains at any given depth *z* were assumed to be equal. Measured settlement in the vicinity of the anode and cathode were different. The measured data shows that measured settlement in the vicinity of the anode was 81 mm, the vicinity of the cathode was 77 mm, the vicinity of the middle position was 90 mm. So, the measured settlement in different position was uneven. However, this is considered to be acceptable because error often appears between the mean analytical and mean measured results.

## Conclusions

The analytical solution using two-dimensional plane model and EKG electrodes were never reached before. Current analytical solutions can’t give relevant guidance for practical engineering. Given the past theoretical derivation, a two-dimensional plane drainage model was proposed for vacuum preloading combined with electro-osmosis consolidation. New EKG electrodes were used in this model. The analytical solution was derived to describe the behavior of the pore water pressure and consolidation in the anode considering the smearing effect. Therefore, the analytical derivation with different two-dimensional models for vacuum preloading combined with electro-osmosis was further developed. Additionally, practical engineering of vacuum preloading combined with electro-osmosis using EKG electrodes can provide a comprehensive reference and guidance.

Based on the developed analytical solutions and measured values, the following conclusions can be drawn. Corrosion and siltation of the EKG were effectively avoided. Mean analytical results and mean measured results were comparable. The changing process of pore-water pressure can be described relatively accurately through the analytical solutions. Applying a vacuum degree of 60 kPa to the soil sample through a vacuum pump, the final mean pore-water pressure obtained from the analytical method was approximately 57.3 kPa. The mean measured pore-water pressure was larger than 60 kPa, which may result from the superposition of vacuum preloading and electro-osmosis. In addition, chemical reaction temperature field changed around the anode; the equal-strain concept caused the difference between the analytical and measured results. A two-dimensional analytical solution considering the free-strain concept was proposed to describe the behavior of pore-water in the following research. The analytical solution and the measured value were more comparable.

## Nomenclature

The following symbols are used in this article:

*i*_h_ Hydraulic gradient

*i*_e_ Electrical gradient

*U*_0_ Effective voltage

*γ*_w_ Specific weight of water

*u* Excess pore water pressure

*u*_s_ Pore-water pressure in the smear zone

$\bar{u}$ Mean pore-water pressure

*w*_0_ Water content

*G*_s_ Proportion

*w*_L_ Liquid limit

*w*_p_ Plastic limit

*k*_1_ Vacuum transfer coefficient

*k*_h_ Hydraulic conductivity

*k*_s_ Hydraulic conductivity of the smear zone

*b*_w_ The width of the EKG drain

*b*_s_ The width of the smear zone for EKG

*B* The width of the model

*k*_e_ Electro-osmotic conductivity

*p*_0_ The value of vacuum preloading

*q*_w,ps_ Well discharge capacity

*l* The depth of the model

*z* The depth of the piezometer

*m*_v_ Volume compression coefficient

*U*_r_ Average degree of consolidation

${\bar{u}}_{f}$ Pore-water pressure in the end of foundation treatment

*t* Time

## Supporting information

S1 Fig Analytical model. (PDF)

S2 Fig Real product of the EKG electrode. (PDF)

S3 Fig Diagram for the EKG electrode. (PDF)

S4 Fig Diagram of model device. (PDF)

S5 Fig Real photo of experiment apparatus. (PDF)

S6 Fig Comparison of analytical result and measures result. (PDF)

S1 Table Basic parameters for the laboratory experiment. (PDF)
