# Supplementary figures and images for: An analytical solution for two-dimensional vacuum preloading combined with electro-osmosis consolidation using EKG electrodes
Source: PLoS One. 2017 Aug 3;12(8):e0180974. doi: 10.1371/journal.pone.0180974 (PMC5542649; doi:10.1371/journal.pone.0180974)

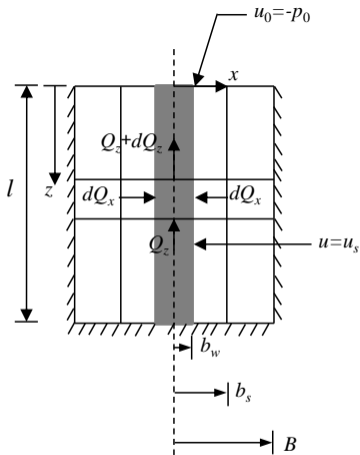

**Fig 1 Vertical cross section of unit cell showing flow condition in vertical drain**

Supplement: S1 Fig — (PDF) [file pone.0180974.s001.pdf]

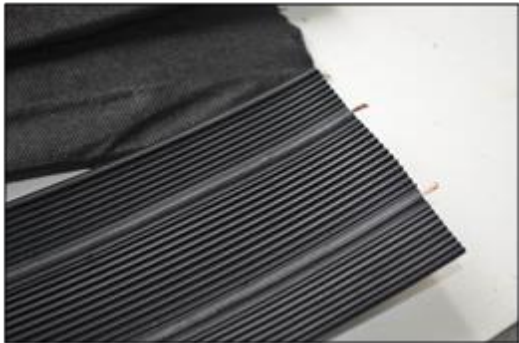

**Fig 2 Real product of the EKG electrode**

Supplement: S2 Fig — (PDF) [file pone.0180974.s002.pdf]

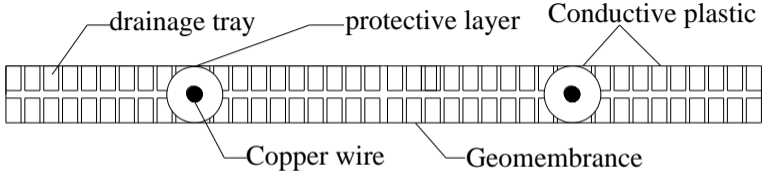

**Fig 3 Diagram for the EKG electrode**

Supplement: S3 Fig — (PDF) [file pone.0180974.s003.pdf]

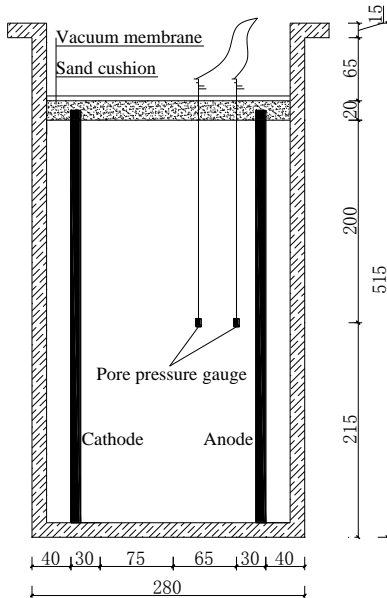

**Fig 4** Diagram of vacuum preloading combined with electro-osmosis model device

Supplement: S4 Fig — (PDF) [file pone.0180974.s004.pdf]
