# Supplementary material for: An analytical solution for two-dimensional vacuum preloading combined with electro-osmosis consolidation using EKG electrodes
Source: PLoS One. 2017 Aug 3;12(8):e0180974. doi: 10.1371/journal.pone.0180974 (PMC5542649; doi:10.1371/journal.pone.0180974)
Supplement: S5 Fig — (PDF) [file pone.0180974.s005.pdf]

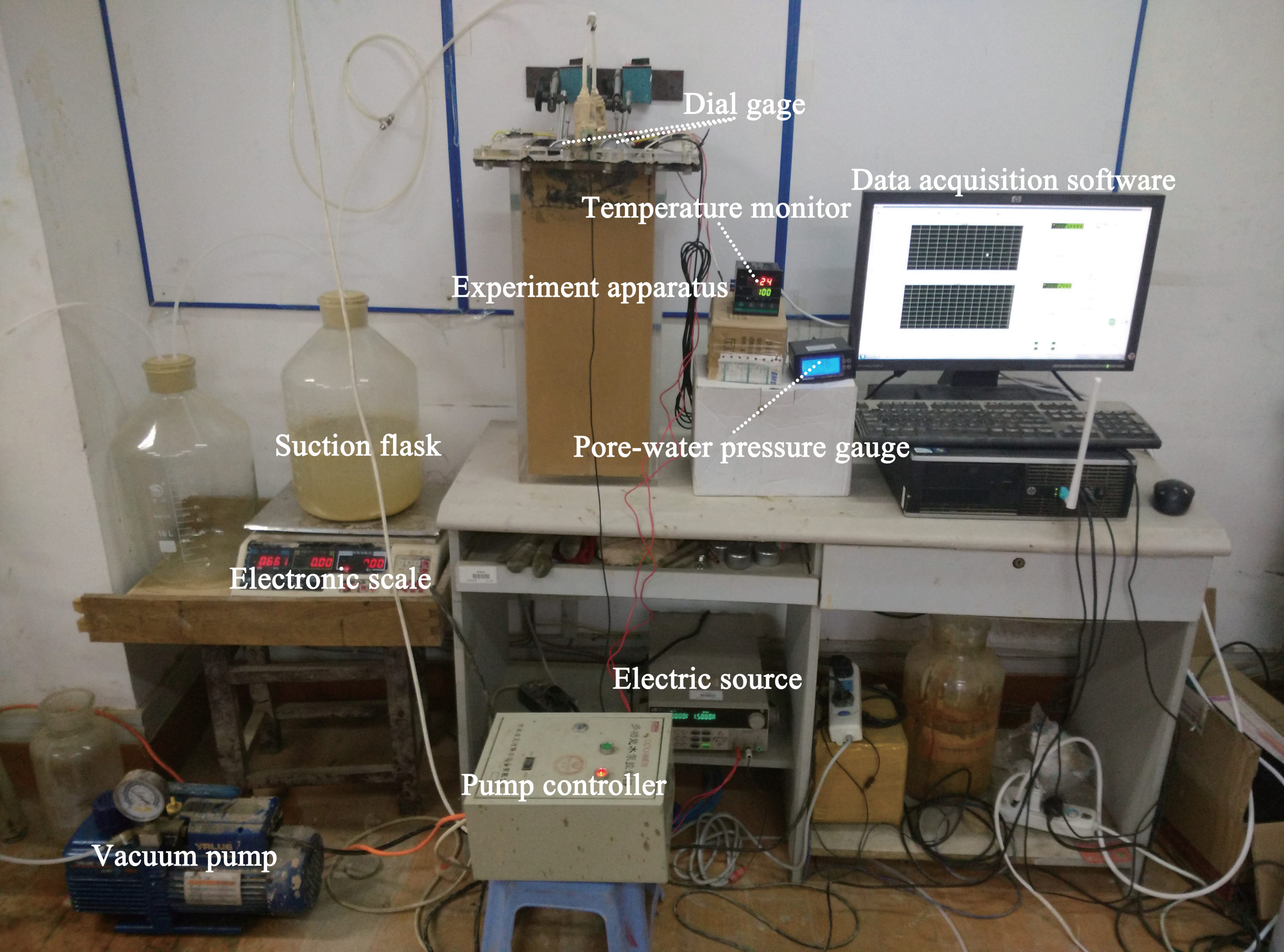
Dial gage
Data acquisition software
Temperature monitor
Experiment apparatus
Suction flask
Pore-water pressure gauge
Electronic scale
Electric source
Pump controller
Vacuum pump
