# Supplementary material for: An analytical solution for two-dimensional vacuum preloading combined with electro-osmosis consolidation using EKG electrodes
Source: PLoS One. 2017 Aug 3;12(8):e0180974. doi: 10.1371/journal.pone.0180974 (PMC5542649; doi:10.1371/journal.pone.0180974)
Supplement: S6 Fig — (PDF) [file pone.0180974.s006.pdf]

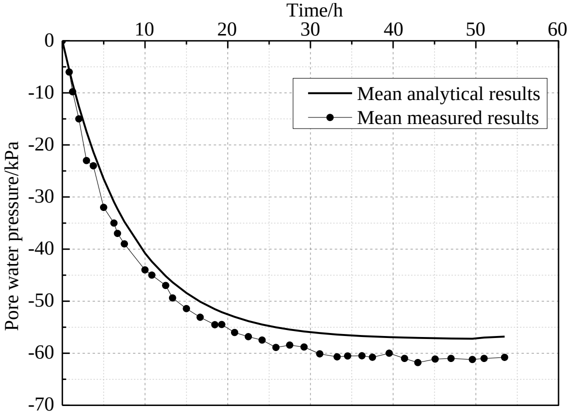


**Fig 5 Comparison of analytical result and measures result regarding dissipation of pore-water pressure**
