# Supplementary material for: An analytical solution for two-dimensional vacuum preloading combined with electro-osmosis consolidation using EKG electrodes
Source: PLoS One. 2017 Aug 3;12(8):e0180974. doi: 10.1371/journal.pone.0180974 (PMC5542649; doi:10.1371/journal.pone.0180974)
Supplement: S1 Table — (PDF) [file pone.0180974.s007.pdf]

Tab 1. Basic parameters for the laboratory test

Parameter	Value	Parameter	Value	Parameter	Value	Parameter	Value
Water content w_0 (%)	50	Plastic index I_p	18.3	Electro-osmotic conductivity k_e (cm^2/sv)	5×10^{-5}	The value of vacuum preloading p_0 (kPa)	60
Proportion G_s	2.75	Clay content (%)	78.6	The width of the EKG drain b_w (mm)	6.5	Well discharge capacity of EKG $q_{w,ps}$ (m^3/s)	18
Liquid limit w_L (%)	42.5	Hydraulic conductivity k_h (cm/s)	4.5×10^{-6}	The width of the smear zone for EKG b_s (mm)	110	The depth of the model l (mm)	400
Plastic limit w_p (%)	24.2	Hydraulic conductivity of the smear zone k_s (cm/s)	1.19×10^{-6}	The width of the model B (mm)	100	The depth of the piezometer z (mm)	200
